# SARS among Critical Care Nurses, Toronto

**DOI:** 10.3201/eid1002.030838

**Published:** 2004-02

**Authors:** Mark Loeb, Allison McGeer, Bonnie Henry, Marianna Ofner, David Rose, Tammy Hlywka, Joanne Levie, Jane McQueen, Stephanie Smith, Lorraine Moss, Andrew Smith, Karen Green, Stephen D. Walter

**Affiliations:** * McMaster University, Hamilton, Ontario, Canada; † Mount Sinai Hospital, Toronto, Ontario, Canada; ‡ Toronto Public Health, Toronto, Ontario, Canada; § Health Canada, Ottawa, Ontario, Canada; ¶ Scarborough Grace Hospital, Ontario, Canada

**Keywords:** SARS, severe acute respiratory syndrome, critical care, risk factors, respiratory protective devices, masks, intubation, nursing, infection control

## Abstract

To determine factors that predispose or protect healthcare workers from severe acute respiratory syndrome (SARS), we conducted a retrospective cohort study among 43 nurses who worked in two Toronto critical care units with SARS patients. Eight of 32 nurses who entered a SARS patient’s room were infected. The probability of SARS infection was 6% per shift worked. Assisting during intubation, suctioning before intubation, and manipulating the oxygen mask were high-risk activities. Consistently wearing a mask (either surgical or particulate respirator type N95) while caring for a SARS patient was protective for the nurses, and consistent use of the N95 mask was more protective than not wearing a mask. Risk was reduced by consistent use of a surgical mask, but not significantly. Risk was lower with consistent use of a N95 mask than with consistent use of a surgical mask. We conclude that activities related to intubation increase SARS risk and use of a mask (particularly a N95 mask) is protective.

Severe acute respiratory syndrome (SARS) was first recognized in Canada in early March 2003 (*1*). Caused by a novel strain of coronavirus, the disease was reported in more than 8,400 people globally, with cases in Asia, Europe, and North America in 2003 (*2*–*4*). SARS is associated with substantial illness and death. The case-fatality rate has been estimated at 13% for patients <60 years and 43% for those >60 years (*5*). In Canada, disease transmission has occurred predominantly among healthcare workers within the healthcare setting (*1*). Preventing SARS transmission to healthcare workers is therefore an important priority (*6*).

Little is known about SARS risk factors for healthcare workers. Determining patient care activities that pose a high risk for infection and possible protective measures for healthcare workers may inform strategies for prevention and may elucidate SARS transmission. Recommended protective equipment for healthcare workers caring for patients with SARS includes a particulate respirator mask (N95) and a goggle or face shield, gown, and gloves (*7*,*8*). One report from Hong Kong has suggested that surgical and N95 masks are protective (9), but few data exist to support the recommendations.

SARS poses a special challenge for healthcare workers who care for the critically ill. Many SARS patients are in critical care units. In a Toronto case series, 29 (20%) of 144 SARS patients were admitted to the intensive care unit (ICU) and 20 (69%) of these 29 received mechanical ventilation (*10*). The close interaction of staff and patients and the nature of invasive patient care activities, such as intubation and other procedures that involve potential exposure to respiratory secretions, raise important questions about the risk for healthcare workers working in critical care units.

To determine risk factors for SARS, we conducted a retrospective cohort study among nurses who worked in two critical care units in a Toronto hospital. We hypothesized that patient care activities (e.g., intubating, suctioning of endotracheal tubes, and administering nebulizers) that increase exposure to respiratory droplets are associated with an increased risk for SARS transmission and that masks protect against infection.

## Methods

### Study Setting and Population

Hospital A is a 256-bed community hospital that provides medical, surgical, obstetric, and pediatric care in the Greater Toronto Area. On March 7, 2003, the 42-year-old son (patient A) of the index patient in the Toronto SARS outbreak (*1*) was seen in the emergency department. He was admitted to the hospital’s 10-bed ICU on March 8. Patient A stayed in the ICU until March 13, the date of his death due to SARS. On March 17, a 77-year-old man (patient B) who had been exposed to patient A in the emergency room on March 7 was admitted to the ICU. He stayed there until his death due to SARS on March 21. Patient C, another emergency room contact of Patient A, was admitted to the hospital’s 15-bed coronary care unit (CCU) on March 13. On March 16, he was transferred to another hospital’s ICU, where he stayed until his death from SARS on March 29. Nurses who worked one or more shifts in hospital A’s ICU from March 8 to 13 and from March 17 to 21 (i.e., when a SARS patient was in the unit) were included in the cohort. Similarly, nurses who worked one or more shifts from March 14 to March 16 in hospital A’s CCU were included.

### Measurements

We recorded the age, sex, and medical history of the nursing staff, including history of any respiratory illness, smoking, conditions that might result in immunosuppression, and use of immunosuppressive medications. Using a standardized data collection form, trained research nurses abstracted information regarding the patient care activities administered by the critical care nurses. To link particular nurses to activities performed in SARS patients’ rooms, we identified nurses’ signatures on patient charts by using a master list of signatures provided by the CCUs. Data collection included type and duration of patient care activities performed. The types of personal protection equipment (goggles, face shield, surgical mask, glove, gown, N95 mask) and the duration and frequency of using the equipment when caring for SARS patients were recorded. Information from the charts was then used to interview nurses about the specific care provided during their shifts. Information provided by the nurses was corroborated whenever possible by data from the charts.

### Case Definition

We used Health Canada’s case definition for suspected or probable SARS cases (*11*). A suspected case was described as fever (>38° C), cough or breathing difficulty, and one or more of the following exposures during the 10 days before onset of symptoms: close contact with a person with suspected or probable SARS, recent travel to an area with recent local SARS transmission outside Canada, recent travel or visit to an identified setting in Canada where SARS exposure might have occurred. A probable case was defined as a suspected SARS case with radiographic evidence of infiltrates consistent with pneumonia or respiratory distress syndrome or a suspected SARS case with autopsy findings consistent with pathologic features of respiratory distress syndrome without identifiable cause. The case definitions are in accordance with the World Health Organization’s clinical case definitions (*12*). All three source patients met the definition for probable SARS cases. For this study, we assessed outcomes for each nurse from the first exposure to a source patient until 10 days (one incubation period) after the last exposure (March 8–April 3 for nurses in ICU and March 14–26 for nurses in CCU). Nurses who met the suspected or probable case definition and the three SARS source patients (patients A, B, and C) were tested for antibodies against SARS-associated coronavirus by immunofluorescence (EUROIMMUN, Luebeck, Germany).

### Statistical Analysis

Fischer’s exact two-sided tests were used to assess risk factors. Exact confidence intervals (CI) were reported. A Kaplan-Meier survival curve was constructed. Data were analyzed by using EpiInfo 2000 (Centers for Disease Control and Prevention, Atlanta, GA) and SAS version 8.0 (SAS Institute Inc., Cary, NC).

## Results

Forty-three nurses worked at least one shift in a critical care unit where there was a patient with SARS; 37 worked in ICU and 6 in CCU. Eight nurses were infected with SARS, four who worked only in the ICU, three who worked only in the CCU, and one ICU nurse who worked one shift in the CCU. All cohort nurses were female; the mean age was 41 years (range 27–65 years). Only two nurses had a history of respiratory illness (one asthma, one bronchitis). Illness onset for the eight nurses was March 16–21. The most common symptoms included fever (8 [100%] of 8), myalgia (7 [87.5%] of 8), cough (6 [75%] of 8) and chills (6 [75%] of 8). Five nurses (62.5%) had headaches, and four (50%) had shortness of breath. Of the eight nurses, four (probable SARS case-patients) had unilateral infiltrates on chest radiograph and four (suspected SARS case-patients) had normal chest radiographs. SARS diagnosis in these eight nurses and in the three SARS source patients was confirmed by serology.

### Patient Care Activities

Relative infection risk for 23 patient care activities is shown in Table 1. None of the 11 nurses who did not enter a SARS patient’s room became ill. Our analysis was thus limited to the 32 nurses who entered a SARS patient’s room at least once. Three patient care activities were associated with SARS infection: intubating (relative risk [RR] 4.20, 95% CI 1.58 to 11.14, p = 0.04); suctioning before intubation (4.20 RR, 95% CI 1.58 to 11.14, p = 0.04); and manipulating an oxygen mask (9.0 RR, 95% CI 1.25 to 64. 9, p ≤ 0.01).

**Table 1:** Relative risk of critical care nurses acquiring SARS by patient care activity^a^

Patient care activity	SARS attack rate (No. cases/No. exposed or unexposed) (%)		Relative risk (95% CI)	p value
	Exposed	Unexposed		
Intubation	3/4 (75)	5/28 (18)	4.20 (1.58 to 11.14)	0.04
Suctioning before intubation	3/4 (75)	5/28 (18)	4.20 (1.58 to 11.14)	0.04
Suctioning after intubation	4/19(21)	4/13(31)	0.68 (0.21 to 2.26)	0.04
Nebulizer treatment	3/5(20)	5/27 (8)	3.24 (1.11 to 9.42)	0.09
Manipulation of oxygen mask	7/14 (50)	1/18 (6)	9.00 (1.25 to 64.89)	0.01
Manual ventilation	2/7 (29)	6/25 (24)	1.19 (0.30 to 4.65)	1.00
Mouth or dental care	5/21 (24)	3/11(27)	0.87 (0.25 to 2.99)	1.00
Insertion of a nasogastric tube	2/6 (33)	6/26 (23)	1.44 (0.38 to 5.47)	0.62
Insertion of an indwelling urinary catheter	2/2 (100)	6/30(0.20)	5.00 (2.44 to 10.23)	0.06
Insertion of a peripheral intravenous catheter	3/5 (60)	5/27 (19)	3.24 (1.11 to 9.42)	0.09
Chest tube insertion or removal	0 (0)	0 (0)		
Insertion of a central venous catheter	2/6 (33)	6/26 (23)	1.44 (0.38 to 5.47)	0.62
Bathing or patient transfer	7/26 (27)	1/6 (17)	1.62 (0.24 to 10.78)	1.00
Manipulation of BiPAP mask	3/6 (50)	5/26 (19)	2.60 (0.8 to 7.99)	0.15
Administration of medication	5/23 (22)	3/ 9 (33)	0.65 (0.20 to 2.18)	0.65
Performing an electrocardiogram	4/12 (33)	4/20 (20)	1.67 (0.51 to 5.46)	0.43
Venipuncture	6/17 (35)	2/ 15 (13)	2.65 (0.63 to 11.19)	0.23
Manipulation of commodes or bedpans	3/5 (60)	5/ 27 (19)	3.24 (1.11 to 9.42)	0.09
Feeding	2/10 (20)	6/22 (27)	0.73 (0.18 to 3.02)	1.00
Debrillation	0/2 (0)	8/ 30 (0.27)		1.00
Cardiopulmonary resuscitation	0/3 (0)	8/29 (28)		0.55
Chest physiotherapy	2/7 (29)	6/25 (0.24)	1.19 (0.30 to 4.65)	1.00
Assessment of patient	6/ 23 (26)	2/ 9 (22)	1.17 (0.29 to 4.77)	1.00
Insertion of peripheral intravenous line	1/1 (100)	7/31 (23)	4.43 (2.31 to 8.50)	0.25
Endotracheal aspirate	3/12 (25)	5/ 20 (25)	1.00 (0.29 to 3.45)	1.00
Bronchoscopy	1/2 (50)	7/ 30 (23)	2.14 (0.46 to 9.90)	0.44
Radiology procedures	4/15(26)	4/17 (24)	1.13 (0.34 to 3.76)	1.00
Dressing change	1/6 (17)	7/26 (27)	0.62 (0.09 to 4.13)	1.00
Urine specimen collected	1/2 (50)	7/30 (23)	2.14 (0.46 to 9.90)	0.44
Fecal specimen collected	0/1 (0)	8/31(26)		1.00
Rectal swab obtained	0/1 (0)	8/31 (26)		1.00
Nasopharyngeal swab obtained	0/2 (0)	8/30 (27)		1.00
Other	2/5 (40)	6/27 (22)	1.80 (0.50 to 6.50)	0.58

^a^SARS, severe acute respiratory syndrome; CI, confidence interval

### Personal Protective Equipment

Use of personal protective equipment and history of high-risk patient care activities among SARS-infected nurses are summarized in Table 2. Relative risk for SARS infection and use of personal protective equipment is summarized in Table 3. Three (13%) of 23 nurses who consistently wore a mask (either surgical or N95) acquired SARS compared to 5 (56%) of 9 nurses who did not consistently wear a mask (RR 0.23, 95% CI 0.07 to 0.78, p = 0.02). The RR for infection was 0.22 (95%CI 0.05 to 0.93, p = 0.06) when nurses who always wore an N95 mask (2 SARS-infected and 14 noninfected nurses) were compared with nurses who did not wear any mask (N95 or surgical mask) consistently (5 SARS-infected and 4 noninfected nurses). The RR for infection was 0.45 (95%CI 0.07 to 2.71, p = 0.56) when nurses who always wore a surgical mask (one SARS-infected and three noninfected nurses) were compared with nurses who did not wear any mask (N95 or surgical mask) consistently (five SARS-infected and four for non-SARS nurses). The difference in RR for SARS infection for nurses who consistently wore N95 masks and those who consistently wore surgical masks was not significant (RR 0.5, 95% CI 0.06 to 4.23, p = 0.5).

**Table 2:** Summary of exposure, personal protective equipment, and participation in high-risk activities of the nurses in whom SARS developed^a^

Nurse	No. of shifts	Location of shift	Total duration of exposure to index patient^b^ (min)	Personal protection used when inside SARS patient’s room	Participation in high risk activities^c^	
1	3	ICU	60	Gown Gloves Surgical mask		
2	3	ICU	385	Gown Gloves N95 Goggles^d^	Intubation, suctioning before intubation	
3	3	ICU^e^	190	Gown^d^ Gloves^d^ N95^d^	Suctioning before intubation	
4	5	ICU	935	Gloves Gown^d^ Goggles^d^ N95^d^	Intubation, suctioning before intubation	
5	3	ICU	555	Gloves Gown N95 Goggles^d^	Intubation	
6	2	CCU	510	None		
7	2	CCU	40	None		
8	2	CCU	510	Gloves^d^		

^a^SARS, severe acute respiratory syndrome; ICU, intensive care unit; CCU, coronary care unit. ^b^Duration of exposure is defined as time spent in a SARS patient’s room. ^c^Intubation, suctioning before intubation. ^d^Indicates that use of this precaution was inconsistent (was not used on one or more occasions). ^e^Nurse 3 worked one shift in coronary care unit.

**Table 3:** Nurses’ risk of acquiring SARS based on use of personal protective equipment^a^

Type of personal protective equipment	Attack rate (%) according to personal protective equipment used		Relative risk (95% CI)	2-Tailed Fisher exact p value
	Consistent	Inconsistent		
Gown	3/20 (15)	5/12 (42)	0.36 (0.10 to 1.24)	0.12
Gloves	4/22 (18)	4/10 (40)	0.45 (0.14 to 1.46)	0.22
N95 or surgical mask	3/23 (13)	5/9 (56)	0.23 (0.07 to 0.78)	0.02
N95^a^	2/16 (13)	5/9 (56)	0.22 (0.05 to 0.93)	0.06
Surgical mask^b^	1/4 (25)	5/9 (56)	0.45 (0.07 to 2.71)	0.56
N95 versus surgical mask^c^	2/16 (13)	1/4 (25)	0.50 (0.06 to 4.23)	0.51

^a^SARS, severe acute respiratory syndrome; CI, confidence interval. ^b^The comparator is use of no mask. The denominator n (total=32) changes for these comparisons as the nurses who consistently used the indicated personal protective equipment were compared to nurses who wore no masks. ^c^Consistent use of the N95 mask versus consistent use of a surgical mask. The denominator n (total=32) changes for these comparisons as the nurses who consistently used the indicated personal protective equipment were compared to the indicated unique group, rather than to the rest of the nurses.

### Time to Event

A Kaplan-Meier curve of the 32 nurses in the cohort who entered a SARS patient’s room is shown in Figure. The figure demonstrates onset of symptoms by number of shifts worked. It shows that if all nurses had worked eight shifts, 53% of them would become infected with SARS. The probability of SARS infection was 6% (8/143) per shift worked.

**Figure:**
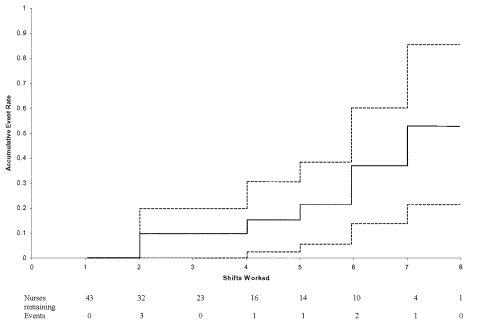
Onset of symptoms for severe acute respiratory syndrome by number of shifts worked (dashed lines represent 95% confidence limits).

## Discussion

We found that critical care nurses who assisted with suctioning before intubation and intubation of SARS patients were four times more likely to become infected than nurses who did not. Manipulation of a SARS patient’s oxygen mask was also a high-risk factor. Our findings support reports that exposure to respiratory secretions or activities that generate aerosols can result in SARS transmission to healthcare workers (*13*).

The 11 nurses in our study who did not enter a SARS patient’s room did not become infected. This finding, along with the finding that respiratory care activities pose high risk, implicates either droplet or limited aerosol generation as a means of transmission to healthcare workers. The finding is compatible with the relative high risk (6% per shift worked) of critical care nurses. Our results did not implicate environmental transmission (i.e., contact through gowns) as a major risk factor. These data are in keeping with the report by Scales and colleagues, in which activities associated with droplet or limited aerosol spread were implicated as important sources of transmission (*14*).

We found a near 80% reduction in risk for infection for nurses who consistently wore masks (either surgical or N95). This finding is similar to that of Seto and colleagues, who found that both surgical masks and N95 masks were protective against SARS among healthcare workers in Hong Kong hospitals (*9*). When we compared use of N95 to use of surgical masks, the relative SARS risk associated with the N95 mask was half that for the surgical mask; however, because of the small sample size, the result was not statistically significant. Our data suggest that the N95 mask offers more protection than a surgical mask.

This study focused on critical care nurses working at the first SARS hospital outbreak in Toronto. Since use of personal protective equipment was not standardized during the study period, it was possible to assess the effect of personal protective equipment. The use of personal protective equipment was highly variable because the nurses were often unaware that their patients had SARS. Our results highlight the importance of using personal protective equipment when caring for SARS patients. We estimate that if the entire cohort had used masks consistently, SARS risk would have been reduced from 6% to 1.4% per shift.

A limitation of this study is that it is retrospective. Recall bias on the part of the critical care nurses is a possibility. We believe that by verifying the information provided (e.g., patient care activities) using medical records, and using the medical records to cue the interviewed nurses, we minimized recall bias. Any prospective evaluation (e.g., using an observer in ICU) after the initial outbreak would have been limited by uniformity in use of personal protective equipment (i.e., use of N95 masks, gowns, gloves, goggles). We acknowledge that the study cohort was small, and this limits inferences that can be made. Nevertheless, these data support current recommendations for use of N95 masks and for special precautions when performing intubations on SARS patients.
